# Assessment of Risk Perception of COVID-19 Post Vaccination amongst the General Population of Riyadh Region

**DOI:** 10.3390/vaccines11071276

**Published:** 2023-07-24

**Authors:** Samia T. Al-Shouli, Nouf O. AlAfaleq, Mohammed Almansour, Munira Alsadhan, Norah Alsalem, Maha Alqahtani, Norah Aldahash, Leena Almazyad, Sadeem Alhazmi, Khaldoon Aljerian

**Affiliations:** 1 Immunology Unit, Department of Pathology, College of Medicine, King Saud University, Riyadh 11461, Saudi Arabia; salshouli@ksu.edu.sa; 2 Department of Biochemistry, College of Science, King Saud University, Riyadh 11451, Saudi Arabia; nalafaleg@ksu.edu.sa; 3 Medical Education Department, College of Medicine, King Saud University, Riyadh 11461, Saudi Arabia; malmansour2@ksu.edu.sa; 4 College of Medicine, King Saud University, Riyadh 11461, Saudi Arabia; 439200450@student.ksu.edu.sa (M.A.); 439200201@student.ksu.edu.sa (N.A.); 439200390@student.ksu.edu.sa (M.A.); 439200262@student.ksu.edu.sa (N.A.); 439200130@student.ksu.edu.sa (L.A.); 439200244@student.ksu.edu.sa (S.A.); 5 Department of Pathology, College of Medicine, King Saud University, Riyadh 11461, Saudi Arabia

**Keywords:** COVID-19, health behaviour, pandemic, risk perception, vaccination, perception

## Abstract

COVID-19 is a highly contagious disease caused by SARS-CoV-2. Vaccination against the virus was first approved in Saudi Arabia in December 2020. Vaccinated individuals are still at risk of getting infected with the virus and can transmit the disease. Therefore, the perception of vaccinated individuals regarding the disease can help limit the spread of the virus. Objectives: To measure the risk perception of COVID-19 following vaccination and factors that have an effect on risk perception; to identify the health protective behaviours of the vaccinated individuals. Methodology: This is a quantitative analytical cross-sectional, questionnaire-based study. The target population includes individuals aged 18 and above who live in the Riyadh region and have been vaccinated, during the period of June 2021 to December 2021. Results: The perception of 30.2% of participants did not change after vaccination, with many participants continuing to “always” take precautions even after vaccination. Numerous factors, such as age, gender, marital status, occupational status, employment status, and total household income, have shown significant effects towards risk perception. Conclusion: Many vaccinated individuals have continued to take precautionary steps and their risk perception has not changed.

## 1. Introduction

Coronavirus disease 2019 (COVID-19) is an infectious disease caused by severe acute respiratory coronavirus-2 (SARS-CoV-2). The first reported discovery of the disease was in Wuhan, Hubei Province, China, in late December 2019 where several patients were suffering from pneumonia of unknown aetiology, which was later sequenced and named coronavirus disease 2019 [1]. Since then, the disease spread rapidly across many countries around the globe, becoming officially recognized as a global pandemic by the world health organization (WHO) in March 2020. As a result, health care systems around the world were overwhelmed by the significant number of patients and there have been enormous losses [2].

Coronaviruses are members of the orthocoronavirinae subfamily of the coronaviridae family. This group of viruses cause symptoms that vary in severity from symptoms resembling the common cold to severe respiratory diseases that is currently seen in COVID-19 [3]. Clinical findings of COVID-19 include fever, cough, fatigue, dyspnoea, and sputum production, arranged in decreasing order from most common to rarest [4]. Transmission of the virus was first identified to be through droplets [5]. However, studies have confirmed that the virus can remain for days on surfaces and remain infectious, which makes transmission by aerosol and fomite possible [6]. As of 11 December 2021, there has been over 267,000,000 confirmed cases and more than 5,000,000 deaths worldwide reported by WHO [7]. The first case of COVID-19 in Saudi Arabia was recorded on 02 March 2020 [8]. Safety measures have included the mandatory quarantine of individuals who have visited affected countries, suspension of schools and universities, enforcing lockdown in cities with high incident number, and limiting travels between cities inside the country [8,9]. On 11 December 2021, the total number of cases in Saudi Arabia was recorded at 550,189 with the total number of deaths equalling 8852 [10]. Continuous effort and research have led to the approval of a few vaccines against the virus. As of 11 December 2021, a total of 48,012,601 vaccines were delivered, including the booster dose. The vaccines, however, do not confer immediate immunity; vaccinated individuals may still be at risk of catching and transmitting the virus [11]. A similar study was conducted by Aw et al. in Singapore, involving 1008 employees across SingHealth Community Hospitals. Over 200 responses were obtained and the prevalence of COVID-19 vaccine hesitancy was found to be 48.5%. It was noted that the prevalence of vaccine hesitancy was elevated within healthcare staff in Singapore during the early stages of the COVID-19 pandemic. The risk factors associated with vaccine hesitancy was found to include females, younger age, method of obtaining COVID-19-related information as from newspapers, and having a known acquaintance free of COVID-19 contact [12]. Risk perceptions were made up of two particular domains: the cognitive domain and the emotional domain: the former relates to people’s understanding of risks—recognized severity and susceptibility; the latter focuses on people’s feelings about the risk, such as worry and anxiety. The rational and cognitive facets of risk perceptions are highlighted in a number of health behaviour theories such as the Health Belief Model (HBM), Protection Motivation Theory (PMT), and the Risk Perception Attitude (RPA) framework [13]. A study conducted in the Netherlands found that “Women reported higher perceptions of risk than men and people with less education expressed more worries about the disease”. However, risk perceptions of a distinct disease are commonly anticipated to have an effect on people’s health behaviours including the uptake of vaccination and precautionary measures [14]. A study conducted in Australia by Adams et al. (2010) found that “The educational status of health professionals had a significant impact on perceptions that are related to COVID-19 vaccine safety. Education is well understood to influence self-care behaviour” [13]. Thus, understanding people’s risk perception of COVID-19 following getting the vaccine plays a significant role in managing the pandemic.

The aim of the current study is to measure the risk perception of COVID-19 in individuals after vaccination and explore the factors that have an impact on it. Furthermore, it is to look at health protective behaviours of vaccinated individuals.

## 2. Methods

### 2.1. Study Design

This is a quantitative analytical cross-sectional questionnaire-based study targeting participants who are 18 years old and above, live in the Riyadh region, and have been vaccinated, during the period of June 2021 to December 2021. Two reliable and validated questionnaires (by Freeman et al. [15] and Shahin et al. [16]) were used following the author’s consent who provided “open access” to these questionnaires (https://drive.google.com/drive/folders/1h4W_C_XtWQcBfQdmrhZpWPX1P-OpcGMg accessed on 15 September 2021).

### 2.2. Pre-Testing

A process of translating and piloting was carried out. Two versions of the questionnaire were created, one in English and one in Arabic. After that, the questionnaires were piloted to improve the quality of the questionnaire, test the clarity, conduct time estimation, and determine the feasibility.

### 2.3. Survey Administration

Samples were collected through the non-probability convenient sampling technique using a link to complete a web-based questionnaire hosted by Google across the Riyadh region from 15 September to 11 October 2021. 

### 2.4. Questionnaire

The questionnaire comprised 30 questions covering five sections: vaccination status, sociodemographic information, knowledge and opinions toward COVID-19 vaccine, sources of information about COVID-19 vaccine, and health protective behaviours. The outcome variable was health protective behaviours that were calculated by adding the score of questions (20–24). As for exposure variables, they were age, gender, nationality, educational level, occupation, health status, employment status, income, marital status, and vaccine type.

### 2.5. Sample Size Computation

The sample size comprised 600 participants, which was calculated using a single proportion formula, N = Zα^2^ P(1 − P)/d^2^, where Zα = 1.96 at a 95% confidence level, *p*-value = 50%, and precision (d) = 4%. When adding the non-response 20% to the original sample size, the total was *n* = 720.

### 2.6. Data Analyses

The data were imported to the Statistical Package for Social Sciences (SPSS) for Windows v.25.0 for analysis. Participants who were younger than 18 years old, did not live in Riyadh, or had not been vaccinated were excluded. Descriptive statistics (mean, standard deviation, frequencies, and percentages) were used to describe the quantitative and categorical variables. As for the inferential tests, one-way ANOVA and independent t-test were performed to determine the significant differences among sociodemographic data. The chi-square test was performed on the cross-tabulation of gender with other sociodemographics. Statistical significance was defined as *p*-value < 0.05. Any unavailable data were considered and dealt with as missing data.

## 3. Results

**Figure 1:**
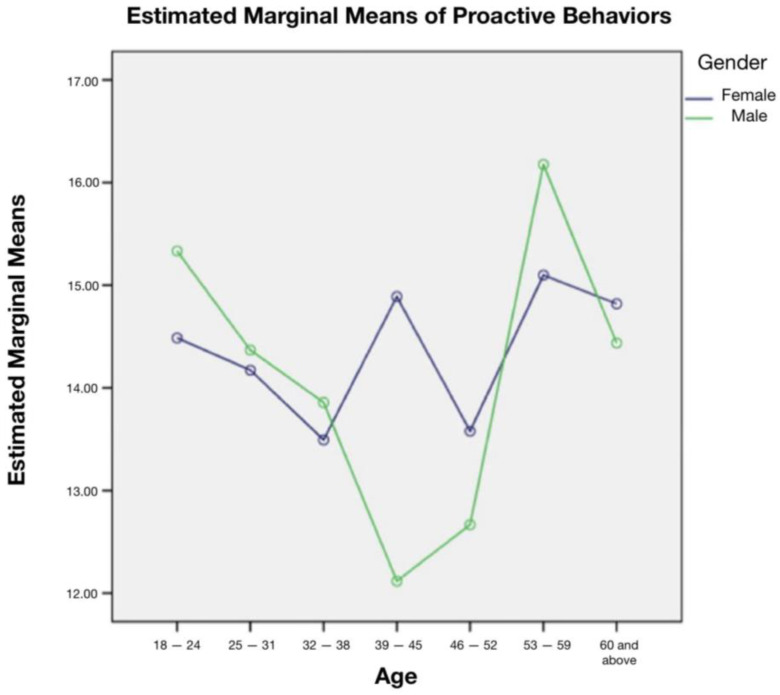
Interaction effect of age and gender on protective behaviour.

The research team received 1010 responses from participants comprising 79.8% females and 20.2% males, aged from 18 years to above 60 years (Table 1). Thirty-four percent (34.4%) of the sample were from 18 to 24 years of age (Table 1). The majority of the participants were of bachelor’s-degree level (56.1%), while high school graduates comprised 25.1% of the total sample (Table 1). Social media exposure can also have an impact in shaping the public’s risk perception. The various platforms together with information processing modes allow an individual to look at or perceive a scenario as the platforms can be relied upon by many as a source of update or information. Here, it was seen that Twitter was widely used for perceiving COVID-19 information. As part of assessing participant awareness, 96.5% of the respondents agreed about the existence of COVID-19 (Table 2). The data showed that 86% had taken the vaccine to protect themselves and their families from the virus. On the other hand, 11.2% took the vaccine just to meet the requirements of work policies, and 2.8% took it to receive permission to travel (vaccine passport) (Table 2). Participants were also asked if they had any worries about getting the infection after receiving the vaccine; 37% answered “sometimes”; 19.6%, 23.6%, 11.1%, and 8.7%, responded with “never”, “occasionally”, “most of the time”, and “always”, respectively (Table 2). Participants were asked if they think that adequate immunity against COVID-19 infection was obtained after receiving COVID-19 vaccine, 35% thought it was “average probability”, 36.4% “high probability”, and 15.6% “very high probability” (Table 2). The risk perception in 30.2% of participants had not changed after getting the vaccine, while the rest changed their perception of “low degree”, “moderate degree”, “high degree”, and “very high degree” with percentages of 5.9%, 21.6%, 24.6%, and 17.7%, respectively (Table 2). The overall mean score for risk perception for vaccinated individuals was 11. The minimum score was 3 and the maximum score was 17, with a standard deviation of 2. Results showed that many participants continued to “always” take precautions even after receiving the vaccine (46.2%) (Table 3). It also showed that most participants, 63.5%, agreed on “always” for frequent hand hygiene and 59.5% believed that wearing masks helps to prevent getting the infection (Table 3). With regard to the effectiveness of social distancing in preventing COVID-19, 59.5% of participants thought it to be “always” effective (Table 3). Factors that have shown significant effect over risk perception (*p*-value 0.05) include age, gender, marital status, occupational status, employment status, and total household income (Table 4). Participants aged between 53 and 59 years had the highest protective behaviour among other age categories with a significant difference where the *p*-value was 0.008. The interaction effect of age and gender on protective behaviour is shown in Table 5 and Figure 1; an effect of age and gender was noted for participants in the 39–45 years age group, where male participants demonstrated a significantly lower average of protective behaviour than female participants.

## 4. Discussion

This study aims to assess people’s risk perception of COVID-19 post vaccination and the demographic factors that can have an influence on it. Vaccinated individuals are still at risk of getting infected with the virus and can transmit the disease; hence, understanding their risk perception and whether they continue taking precautionary measures or not can help to control the spread of the virus to others. An overwhelming majority of the participants have a positive reflection of scientific facts on COVID-19, preventive health measures, and vaccination. This study has shown that the risk perception toward COVID-19 in 30.2% of participants did not change after getting the vaccine and 46.20% continued to “always” take precautions even after the vaccine. Participants changed their perception of “low degree”, “moderate degree”, “high degree”, and “very high degree” with percentages of 5.9%, 21.6%, 24.6%, and 17.7%, respectively. The demographic factors that showed a significant effect over risk perception in the current study were age, gender, marital status, occupational status, employment status, and total household income. 

A study by Shahin et al. showed that Saudi Arabian participants conveyed significantly higher mean scores regarding the prospect of contracting COVID-19 in the absence of preventive measures compared with participants from Egypt and Jordan. This reflected a generally higher perception of susceptibility and anxiety to COVID-19 for respondents from Saudi Arabia [1,16]. 

A study conducted by Alsaif et al. showed that 69.2% of participants had a positive attitude toward prevention of COVID-19 and 68% presented a good practice regarding preventing the spread of the infection. The study further assessed the level of knowledge, showing 59% presenting a good level of knowledge toward COVID-19 prevention [17].

The preceding results have been found to be directly proportional to the age of the participants in the study. Both employment and marital statuses were also factors that influenced the level of knowledge and the practice of participants toward COVID-19, where the employed and elderly showed a significant positive attitude and practice for COVID-19. These results coincide with the results of another study that also attempted to assess the knowledge, attitude, and practice toward preventing COVID-19 among the Hail Community, Kingdom of Saudi Arabia. The only factor that showed a significant association with the results of this study was gender; male participants showed a slight increase (60%) in the level of awareness compared to female participants (57%). However, with regard to the practice toward COVID-19 infection, female participants showed a slightly better practice (71.5%) than male (64.6%) [17]. 

Previous studies on the perception of COVID-19 and the attitude toward prevention reported a good level of awareness with a positive attitude. The results were directly proportional to factors such as age, employment, and social status of the participants, which corresponded with this study. The correlation between gender and level of knowledge was also seen to be parallel with the current study; gender had an influence over risk perception [2,3,18,19].

With the growing use of social media platforms among people of all ages these days, these platforms have been used by many as the main source of information regarding all sorts of topics. Whether it is WhatsApp, Twitter, or Facebook, all platforms were being flooded with news and alleged facts during the emergence of COVID-19. As stated in this study, Twitter was found to be the most common source used for information about COVID-19 (44.95%). Jaber et al. were concerned about the same objective and evaluated the awareness and risk perception of 3167 participants from Iraq and Jordan [20]. They concluded that more than 60% of all participants have relied on medical staff for information about the virus, whereas social media was the second most common source of information related to COVID-19. The results showed that both social media platforms and medical staff were the main sources of information from which respondents received COVID-19-related knowledge. Knowing that social media is being used by many as a reference of knowledge, it should be used by authorities to spread informative and accurate facts related to the virus and the correct ways of protection. 

According to the results of the current study, protective measures like frequent handwashing, wearing masks, and social distancing were being followed by most participants and are believed to be effective against COVID-19. Similar results were found by Siddiqui et al. in which 443 individuals from Saudi Arabia were surveyed [21]. The survey showed that 356 (84%) believed that washing hands was an effective measure of protection against the infection, whilst 333 respondents (79%) agreed that keeping a safe distance could limit the spread of the virus. The results that were influenced by the level of education of the participants had an effect on their choice of practice to protect themselves from COVID-19. The data retrieved from this study can be used by authorities to improve trust between the ministry of health and people. Data can also be used to target the platforms most used by the population and increase their awareness through it. 

**Figure 2:**
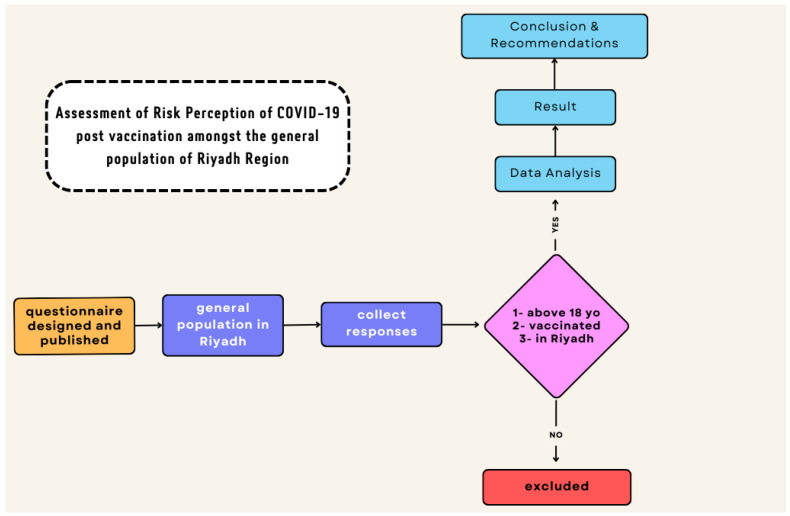
Flowchart of the study methodology.

This study is not without limitations; certain limitations of the study have been identified that, in future studies, can be addressed to augment the unique findings of this study. This study was limited to only the Riyadh region and was conducted neither at a national nor a regional level; the findings may not represent all the Saudi citizens from all regions and therefore cannot be generalized. However, it was conducted on a sufficient number of Riyadh citizens. Additionally, the data of the research are slightly outdated as the information was collected during the pandemic. Vaccinated individuals aged above 18 years were looked at; unvaccinated individuals, those aged less than 18 years, and those who received booster doses were not included (Figure 2). Furthermore, the presence of chronic illnesses or immunodeficiency diseases and their contribution to the individuals’ perception and adherence to health protective behaviours were not included in this study. Gender response can also be seen as a limitation; the majority of respondents in this study were females. This study did not address the risk perception toward COVID-19 of individuals prior to receiving the vaccine; therefore, it is recommended that future studies compare the risk perception of the individual toward the disease prior to and after receiving the vaccine. Furthermore, this is a questionnaire-based study and, like other research using questionnaires, several limitations and biases could be encountered, including misunderstood questions; responders may have a hidden agenda, lack of personalization, and questionnaire fatigue.

## 5. Conclusions/Recommendations

This study has shown that an overwhelming majority of the participants have a positive reflection of scientific facts on COVID-19, preventive health measures, and vaccination. This may be attributed to the highly educated population and their choice to respond positively to reliable sources. There are current limitations in the literature on the risk perception of COVID-19 after vaccination, particularly in our region. Our study helps to address and fill this limitation by assessing the risk perception of COVID-19 amongst the general population in the Riyadh region after receiving the COVID-19 vaccine. 

Given the urgency of this issue and the significance of the results herein, we suggest similar studies to be conducted at both the national and regional levels. We recommend future studies to involve unvaccinated individuals, those aged below 18 years, and those who have received booster doses.

## Figures and Tables

**Table 1:** Cross-tabulation of gender with other sociodemographics.

Item	Response	Gender, *n* (%)		Total
		Female	Male	
Age	18–24	293 (29.1)	54 (5.4)	347 (34.4)
	25–31	58 (5.7)	19 (1.9)	77 (7.6)
	32–38	76 (7.5)	14 (1.4)	90 (8.9)
	39–45	108 (10.7)	17 (1.7)	125 (12.4)
	46–52	128 (12.7)	33 (3.3)	161 (15.9)
	53–59	81 (8.04)	34 (3.4)	115 (11.4)
	60 and above	61 (6.05)	32 (3.2)	93 (9.2)
Nationality	Saudi	789 (79.2)	196 (19.4)	994 (98.6)
	Non-Saudi	7(0.7)	7(0.7)	14 (1.4)
Marital Status	Single	327 (32.4)	72 (7.1)	399 (39.6)
	Married	430 (42.7)	128 (12.7)	558 (55.4)
	Widowed	26 (2.6)	1 (0.1)	27 (2.7)
	Divorced	22 (2.2)	2 (0.2)	24 (2.4)
Parental Status	Have children	420 (41.7)	120 (11.9)	540 (53.6)
	Do not have children	110 (10.9)	18 (1.8)	128 (12.7)
	The question is not applicable to me	268 (26.6)	61 (6.05)	329 (32.6)
	Rather not to answer	7 (0.7)	4 (0.4)	11 (0.9)
Highest level of education	Elementary School	4 (0.4)	0	4 (0.4)
	Middle School	15 (1.5)	9 (0.9)	24 (2.4)
	High School	253 (25.1)	35 (3.5)	288 (28.6)
	University bachelor	465 (46.1)	100 (9.9)	565 (56.1)
	University Masters	44 (4.4)	41 (4.1)	85 (8.4)
	University PhDs or equivalent	9 (0.9)	16 (1.6)	25 (2.5)
	The question is not applicable to me	15 (1.5)	2 (0.2)	17 (1.69)
Employment Status	Student	284 (28.2)	53 (5.3)	337 (33.4)
	Full time job	216 (21.4)	81 (8.04)	297 (29.5)
	Part time job	26 (2.6)	3 (0.3)	29 (2.9)
	Self empolyed	9 (0.9)	18 (1.8)	27 (2.7)
	The question is not applicable to me	270 (26.8)	48 (4.7)	318 (31.5)
Total household income	Less than 1333$	27 (2.7)	5 (0.5)	32 (3.2)
	1334–2666$	112 (11.1)	7 (0.7)	119 (11.8)
	2667–5333$	155 (15.4)	43 (4.3)	198 (19.6)
	5334–7999$	118 (11.7)	41 (4.1)	159 (15.8)
	8000–10,666$	52 (5.1)	18 (1.8)	70 (6.9)
	10,667$ and more	83 (8.2)	46 (6.5)	129 (12.8)
	Rather not to disclose	258 (25.6)	43 (4.3)	301 (29.9)

**Table 2:** Distribution of participant’s risk perception responses and their level of knowledge toward COVID-19 infection after receiving COVID-19 vaccination.

Item	Response	*n*	%
Do you believe that COVID-19 exists	Yes	975	96.5%
	No	35	3.5%
What is the main reason for your willingness to receive the COVID-19 vaccine	To protect myself/my family from infection	869	86%
	To meet the requirements of work policies	113	11.2%
	To receive a permission to travel (vaccine passport)	28	2.8%
I have worries about getting COVID-19 infection after receiving the vaccine	Sometimes	374	37%
	Occasional	238	23.6%
	Never	198	19.6%
	Most of the time	112	11.1%
	Always	88	8.7%
After receiving COVID-19 vaccine, adequate immunity against COVID-19 infection is obtained	High probability	368	36%
	Average probability	354	35%
	Very high probability	158	15.6%
	High Not getting enough immunity	76	7%
	Low propability	54	5.4%
My risk perception towards COVID-19 has increased in comparison to before I received the COVID-19 vaccine	My perception hasn’t changed	305	30.2%
	High degree	248	24.6%
	Moderate degree	218	21.6%
	To a very high degree	179	17.7%
	Low degree	60	5.9%

**Table 3:** Distribution of study participants’ response toward their health protective behaviours.

Item	Response	*n*	%
I continue to take precautions after receiving COVID-19 vaccine	Never	15	1.5%
	Occasional	31	3.1%
	Sometimes	179	17.7%
	Most of the time	318	31.5%
	Always	467	46.2%
You might be infected with Coronavirus (COVID-19) in the future if you do not take any preventive measures	Improbable	10	1%
	Weakly probable	47	4.7%
	Moderately likely	210	20.8%
	Very likely	381	37.7%
	Extremely likely	362	35.8%
Frequent hand hygiene helps to prevent Coronavirus (COVID-19)	Never	6	0.6%
	Occasional	14	1.4%
	Sometimes	74	7.3%
	Most of the time	275	27.2%
	Always	641	63.5%
Wearing masks help to prevent Coronavirus (COVID-19)	Never	5	0.5%
	Occasional	11	1.1%
	Sometimes	84	8.3%
	Most of the time	308	30.5%
	Always	602	59.6%
Keeping social distancing helps to prevent Coronavirus (COVID-19)	Never	5	0.5%
	Occasional	16	1.6%
	Sometimes	83	8.2%
	Most of the time	305	30.2%
	Always	601	59.5%

**Table 4:** Univariate analysis of health protective behaviour scores in relation to the characteristics of study participants.

Item	Response	Mean ± SD	*p*-Value
Age	18–24	14.6 ± 4.3	0.008 *
	25–31	14.2 ± 4.7	
	32–38	13.5 ± 4.6	
	39–45	14.5 ± 4.8	
	46–52	13.4 ± 5.1	
	53–59	15.4 ± 4.1	
	60 and above	14.7 ± 5.3	
Gender	Female	14.4 ± 4.5	0.853
	Male	14.4 ± 5.1	
Nationality	Saudi	14.4 ± 4.6	0.410
	Non-Saudi	13.4 ± 5.1	
Marital Status	Single	14.6 ± 4.5	0.363
	Married	14.2 ± 4.8	
	Widowed	15.2 ± 5.1	
	Divorced	15.1 ± 4.2	
Parental Status	Have children	14.2 ± 4.9	0.469
	Do not have children	14.3 ± 4.4	
	The question is not applicable to me	14.7 ± 4.5	
	Rather not to answer	13.3 ± 3.9	
Highest level of education	Elementary School	18.2 ± 1.5	0.264
	Middle School	14.6 ± 14.6	
	High School	14.4 ± 4.5	
	University bachelor	14.3 ± 4.6	
	University Masters	13.7 ± 5.1	
	University PhDs or equivalent	15.7 ± 5.0	
Employment Status	Student	14.6 ± 4.4	0.682
	Full time job	14.4 ± 4.8	
	Part time job	14.2 ± 4.7	
	Self empolyed	13.9 ± 6.2	
Total household income	Less than 1333$	15.5 ± 4.6	0.399
	1334–2666$	14.1 ± 4.5	
	2667–5333$	14.6 ± 4.4	
	5334–7999$	14.8 ± 5.02	
	8000–10,666$	14.04 ± 4.6	
	10,667$ and more	13.8 ± 4.7	
	Rather not to disclose	14.2 ± 4.7	

* Significant at 0.05.

**Table 5:** Interaction effect of age and gender on protective behaviour.

	Mean ± SD
Female	
18–24	14.5 ± 4.3
25–31	14.2 ± 4.3
32–38	13.5 ± 4.7
39–45	14.9 ± 4.8
46–52	13.6 ± 5.01
53–59	15.1 ± 3.9
60 and above	14.8 ± 4.9
Male	
18–24	15.3 ± 4.3
25–31	14.4 ± 5.9
32–38	13.8 ± 4.5
39–45	12.1 ± 4.5
46–52	12.7 ± 5.4
53–59	16.2 ± 4.5
60 and above	14.4 ± 6.1

## Data Availability

Data is unavailable due to privacy or ethical restrictions.
